# African civil society initiatives to drive a biobanking, biosecurity and infrastructure development agenda in the wake of the West African Ebola outbreak

**DOI:** 10.11604/pamj.2016.24.270.8429

**Published:** 2016-07-27

**Authors:** Akin Abayomi, Sahr Gevao, Brian Conton, Pasquale Deblasio, Rebecca Katz

**Affiliations:** 1 Global Emerging Pathogens Treatment Consortium (GET), Division of Haematology, Faculty of Medicine and Health Sciences, Stelenbosch University and the National Health Laboratory Service of South Africa, Tygerberg Hospital, Cape Town, South Africa; 2 Global Emerging Pathogens Treatment Consortium (GET), College of Medicine and Allied Health Sciences, University of Sierra Leone, Sierra Leone; 3 Global Emerging Pathogens Treatment Consortium (GET), Physio-Fitness rehabilitation Centre, Freetown, Sierra Leone; 4 Global Emerging Pathogens Treatment Consortium (GET), Integrated Systems Engineering Srl, Milan, Italy; 5 Milken Institute School of Public Health, George Washington University, Washington DC, USA

**Keywords:** Biobanking, biosecurity, infrastructure, consortia

## Abstract

This paper describes the formation of a civil society consortium, spurred to action by frustration over the Ebola crises, to facilitate the development of infrastructure and frameworks including policy development to support a harmonized, African approach to health crises on the continent. The Global Emerging Pathogens Treatment Consortium, or GET, is an important example of how African academics, scientists, clinicians and civil society have come together to initiate policy research, multilevel advocacy and implementation of initiatives aimed at building African capacity for timely and effective mitigations strategies against emerging infectious and neglected pathogens, with a focus on biobanking and biosecurity. The consortium has been able to establish it self as a leading voice, drawing attention to scientific infrastructure gaps, the importance of cultural sensitivities, and the power of community engagement. The GET consortium demonstrates how civil society can work together, encourage government engagement and strengthen national and regional efforts to build capacity.

## Commentary

### Introduction

The 2014-2015 Ebola virus disease outbreak devastated an already weak public health infrastructure in West Africa. Ebola deaths amongst African healthcare workers only exacerbated the problem. The most affected nations had to rely on international response efforts to mitigate the consequences of the disease; a response that came many months too late. It became apparent that a unified, indigenous voice or coalition of African experts to assist with early detection, defining and coordinating the logistics of response on the ground was lacking. This paper describes the formation of a civil society consortium, spurred to action by frustration over the Ebola crises, to facilitate the development of infrastructure and frameworks including policy development to support a harmonized, African approach to health crises on the continent. The Global Emerging Pathogens Treatment Consortium, or GET, is an important example of how African academics, scientists, clinicians and civil society have come together to initiate policy research, multilevel advocacy and implementation of initiatives aimed at building African capacity for timely and effective mitigations strategies against emerging infectious and neglected pathogens. The GET consortium demonstrates how civil society can work together, encourage government engagement and strengthen national and regional efforts to build capacity consistent with the Global Health Security Agenda.

### Establishment of GET

**Figure 1:**
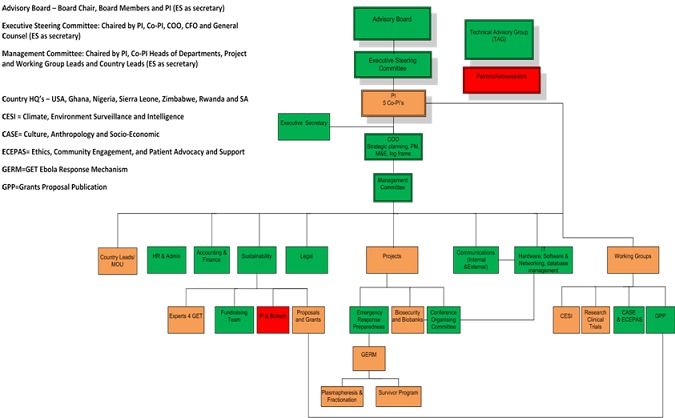
GET organizational structure

The GET consortium was established on the 21^st^ of August 2014 during the peak of the Ebola outbreak and soon after the index case arrived in Lagos, the 23 million population mega city and commercial capital of Nigeria. The consortium's, aim is to bring like-minded and relevant experts together, who are working in Africa to provide indigenous capacity to collectively address an ever increasing menace of emerging and re-emerging dangerous infectious pathogens. As the Ebola outbreak unfolded in Lagos, it became apparent that an unexpected percentage of Ebola patients were surviving. The founding members of GET, amongst other initiatives, prioritized their mission to find an immediate, local solution to counter the spread of Ebola for which there is still no known treatment-particularly in a vulnerable mega city like Lagos. It was hypothesized based on very rudimentary evidence and animal studies, that if the outbreak was not contained, there were going to be an increasing number of survivors and possibly the only immediate solution was the use of passive immunity from survivors using convalescent plasma donations containing anti Ebola antibodies and transfused under stringent conditions to new cases [1, 2]. This approach became a collaborative effort with international partners, aimed at building the regional capacity to harvest, process and submit convalescent plasma to clinical trials during the outbreak. This eventually led to the convalescent trial capacity drive in Liberia, Sierra Leone, Guinea and Nigeria, which is still on going. This was also accompanied by community outreach to survivors as well as developing guidelines and advice on the ethics of research during public health crises. To achieve the objectives of the convalescent approach, the founding GET consortium members reached out to their colleagues across the region, and in particular within Guinea, Sierra Leone and Liberia, to see how this convalescent plasma strategy could be accelerated. The recruitment drive through person to person contact and existing networks of related Africa consortia soon extended across the whole continent with enthusiastic response from professionals from a variety of disciplines offering of their time voluntarily. The network now includes experts from infectious diseases, pathology, bio-informatics, bio-banking, ethics, social science, economics, patient advocacy, logistics, engineers, ecologists, government administrators, creating artist and several other disciplines. A temporary governance structure was developed and officers appointed. The consortium was registered in Nigeria, Ghana and the United States. The GET consortium ultimately intends to register a presence in every African country as well as in major hubs internationally to ensure that the consortium taps into the wider African Diaspora, while also reaching out to international scientists and supporters who have an interest in capacity development on the African Continent. Through initial institutional funding provided by the Bill and Melinda Gates foundation, GET was able to sustain its growth and develop into a fully functional Civil Society Organization aimed at public health policy research and implementation with expanding impact across several sectors of medicine, logistics and the social sciences. Once GET was formally established, the consortium identified a series of priority issues and proposals for immediate action. These priority issues were driven by the fragmented response to the Ebola outbreak, which appeared to lack sensitivity to the cultural and indigenous knowledge based systems of the continent. This resulted in the formation of nine policy and operational working groups (Figure 1). These include: Ethics, Community Engagement, Patient Advocacy, and Support (ECEPAS); Research and Clinical Trials; Plasmapheresis; Grant, Proposal and Publications; Bio-banking, bio-data, bio-safety, biosecurity, bioinformatics (B5); Cultural, Anthropological, Social and Economic Impact (CASE); HIV cure; Climate, Environmental and Disease Surveillance and bio-intelligence (CESI); Media and Communication. Through the executive committee and the working groups, the GET consortium has been able to move rapidly to organized academic partners, governments, international organizations and associated partners.

### Results

In a relatively short period of time, the civil society consortium has been able to forge new partnerships, establish regional positions, and facilitate commitments by governments. Table 1 depicts some of the GET organized events, proposals, and workshops. In collaboration with West African regional partners, GET was instrumental in organizing the first African Voices and Leadership conference on Ebola held in Dakar in January 2015. The meeting brought together representatives from leading regional and continental health authorities (including the United Nations, World Food Programme, World Health Organization, African Union, MSF, ECOWAS, WAHO, NEPAD, and MSF), representatives of the ministries of health of the ECOWAS region, scientists, Ebola survivors and Ebola treatment center health care givers. During this meeting the multitude of socio-economic, infrastructural and capacity deficiencies that permitted the Ebola outbreak to affect so many, were identified and crystalized into what became the **Dakar Declaration** [3]. This meeting clearly defined key infrastructure deficiencies, such as biobanking facilities and biosecurity infrastructures, and the lack of a framework to address the stigma, rehabilitation and care of Ebola survivors. The necessary policies that would govern such infrastructure development and frameworks required urgent attention. GET has facilitated a series of meetings to explore hosting a conference focused on biobanking and biosecurity infrastructure, and has worked to establish African infrastructure to conduct plasmapheresis and plasma storage facilities in key strategic locations in Africa to support Ebola clinical trials. The success of GET can also be demonstrated in the consortium's interventions in Sierra Leone. GET has provided templates and expertise in fair contract negotiations for clinical trials agreements to ensure intellectual property rights are maintained for the Ebola affected countries. GET also led the drive to ensure the Ebola affected countries were aware of the biosecurity and biosafety threats posed by Ebola samples, given the insecure environments of the current laboratory systems, and provided options to take ownership and control of the samples. The samples left over from the 2014/15 Ebola outbreak were strewn throughout partner labs that were set up all over the subregion. However some of these labs have shut down. More are due to do so, and there is as yet no agreed process regulating safe handing over or disposal of dangerous substances. These remaining samples of Ebola are left without the requisite conventional security to prevent their accidental discharge or intentional theft for unregulated research or possible non-peaceful activities. GET has been entrusted with raising the Government awareness on biobanking, drafting a national policy, and crafting the road map for data rescue of the samples belonging to Sierra Leone with a view to having a safe and sustainable biobank GET used established networks to access the membership of relevant African Societies and established academic research networks such as the H3Africa and B3Africa consortia to build upon established guidelines for sample and data management as the basis for formulating standard operating practices in the Ebola affected countries. These examples illustrate how increased technical expertise, as promoted and shared by GET, enabled south-south collaboration. In addition to these activities organized or supported by GET, the consortium has improved its regional and global recognition as a representative voice committed to improving infrastructure and capacity to cope with public health crises caused by epidemics in Africa. Part of this recognition has been the consistent invitations to participate in and speak at regional, international conferences and policy forums, becoming an important voice in policy discussions around global health security, biosafety, biosecurity, biobanking and ethics. GET is building up a number of reports and manuscripts that document its experience on this trajectory and these can be found on its website [4].

**Table 1:** GET activities

Name	Date	Outcome
Ethics, Community Engagement and Ebola Survivor Advocacy and Support	October 2014	Ethical review of protocol for first ever convalescent trial in Liberia
Lagos State Ministry of Health	October 2014	Through an MOU with the Lagos State Government Ministry of Health, GET has supported the Ebola Core Research group formed in Lagos
Logistical support for ClinicalRM	November 2014	Logistics for massive air drop of blood mobiles, apheresis machines and medical supplies to Guinea Liberia and Nigeria
Biobanking Proposal	January 2015	First indigenous initiative proposal for a comprehensive attempt to secure all Ebola samples in the region and develop biobanking and biosafety facilities and frameworks presented to the Global Partnership
First African Voices and Leadership Conference on Ebola and EID	January 2015, Senegal	Dakar Declaration: Lack of biobanking and biosecurity capacity and frameworks is contributing factor to rapid spread of West African Ebola outbreak
Ebola Survivor Workshop with Ebola Survivors Association of Liberia (ESAL)	January 2015, Liberia	Growth of 500 to 800 survivors registered with ESAL
Ebola Survivor Workshop with Sierra Leone Association of Ebola Survivors (SLAES)	February, 2015 Freetown, Sierra Leone.	Building network of Ebola survivors and infrastructure. Piloted creative fine art as a means of healing Post Traumatic Stress Disorder experienced by survivors.
Knowledge, Attitude and Perception Study	February 2015, Sierra Leone	Introduced new study to attempt to understand factors in community that generate discrimination against Ebola survivors.
Engagement with NOGUCHI BSL 3 Facility	August 2015, Accra, Ghana	Development of Memorandum of Understanding for support to the most affected countries.
1^st^ WHO meeting on Ebola and Biobanking	May 2015, Geneva, Switzerland	GET represented at first WHO meeting on Ebola and biobanking. Agreed that an accelerated approach to biobanking was required with more indigenous representation to be scheduled in the West African region.
2^nd^ WHO meeting on Ebola survivors and Biobanking	August 2015 Freetown, Sierra Leone,	A continuation of the discussion initiated in Geneva with a more focused approach on indigenous solutions. This was immediately followed by a GET workshop on Biobanking and Biosecurity
National biobanking and biosecurity policy workshop in Sierra Leone	August 2015 Freetown, Sierra Leone	First indigenous workshop on biobanking and biosecurity, aimed at supporting the Government of Sierra Leone to develop its national biobanking and biosecurity framework.
West African Task force for the research into Emerging and re-emerging Infections (WATER).	August 2015 Sierra Leone	Founding members of GET and representatives of WATER agreed to synchronize activities in the West African Region, to ensure elimination of duplicity and ensure effective use of resources and human capacity.
MSF-GET	August 2015 Sierra Leone	Series of meeting between GET and MSF culminated in a strategic approach to coordinate and support the efforts of GET and WATER to accelerate biobanking and biosecurity capacity in Africa
African Gong	August 2015 Cape Town, South Africa	Founding members of African Gong and GET met to map out the modalities of hosting the first African Conference on Science Communication and Public understanding of science.
WHO Ethics meeting	September 2015 Cape Town, South Africa	GET PI gave a summary of the second WHO meeting held in Freetown, aimed at further refining guidelines for the ethical approach to public health emergencies based on lessons learned during the Ebola outbreak.
Trends Symposium to support the Biological Weapons Convention	September 2015 Poland	GET Principal Investigator was requested to give an overview of the biosecurity issues in the Ebola affected region and how new technology has made a difference.
Royal College of Pathologists of the United Kingdom (RCPath)	September 2015 Kampala, Uganda	GET and senior members of the International Division of the Royal College of Pathology, met at the Labskills Africa conference to map out future collaborations
Global Health Security Agenda (GHSA) meeting organized by the African Society of Laboratory Medicine (ASLM).	October 2015. Freetown, Sierra Leone.	GET members invited, participated in this meeting contributing to improved understanding of the biosecurity deficits in the region.
Africa Union Peace and Security Department. Consultative meeting on the Universalization of the Biological Weapons Convention (BWC) in Africa.	October 2015. Addis, Ethiopia	GET was invited as a speaker and observer to share experiences in the containment strategies of the Ebola Outbreak and contributions towards capacity building for enhanced implementation of the BWC.

### Discussion

The GET consortium has been able to establish itself as a leading voice, drawing attention to scientific infrastructure gaps, the importance of cultural sensitivities, and the power of community engagement. These efforts are vital to the core mission of the Global Health Security Agenda-making the world safer from infectious disease threats. The GET consortium is working to build African capacity to detect, respond and recover from infectious diseases, and doing so through their ability to harness the training, passion and capacity of African medical and non-medical professionals. The GET consortium can be a model for all regions of the world, demonstrating that committed civil society can engage in the hardest problems of their region in support of governments and populations. The consortium, however, has faced multiple challenges. Potential research collaborators have questioned the strength of the consortium, and even questioned the possibility of African professionals to desire to come together as they have under GET, complicating fund raising efforts. GET realizes that it should also turn its attention to indigenous funding streams including corporate social responsibility to accelerate the African bio-economy. There are many health related challenges across the African continent due to endemic burden of disease and a multitude of environmental and host factors that facilitate the emergence of biological threats. These health challenges are amplified by an insufficient clinical workforce, infrastructure challenges, strained governments, and limited resources. In the midst of this challenging environment, the GET consortium has emerged as a means for African civil society to work together to support improved health outcomes, strengthen global health security, and build much needed capacity to combat biological threats.

## References

[1] Mupapa K, Massamba M, Kibadi K, Kuvula K, Bwaka A, Kipasa M, Coleburnders R, Muyembe-Tamfum JJ (1999). Treatment of Ebola Hemorrhagic Fever with Blood Transfusions from Convalescent Patients. Journal of Infectious Diseases..

[2] Dye JM, Herbert AS, Kuehne AI, Barth JF, Muhammad MA, Zak SE, Ortiz RA, Prugar LI, Pratt WD (2012). Postexposure antibody prophylaxis protects nonhuman primates from filovirus disease. Proceedings of the National Academy of Sciences..

[3] (2015). African Voice and Leadership Meeting to Accelerate the Evaluation of Potential Treatments and Vaccines for Ebola in West Africa. Dakar Declaration.

[4] http://www.getafrica.org.

